# Personal

**Published:** 1901-02

**Authors:** 


### PERSONAL.

Dr. Charles A. L. Reed, of Cincinnati, O., president of the American Medical Association, spent a day or two in Buffalo during the latter part of January. He was the guest of Dr. William Warren Potter and was en route to Albany to attend the ninety-fifth annual meeting of the Medical Society of the State of New York.

---

Dr. George T. Stevens, of New York, spent a few days in Buffalo recently and during his visit delivered an address before the Section on Ophthalmology, etc., of the Buffalo Academy of Medicine, the title of which is noted elsewhere. Dr. and Mrs. Frank Whitehill Hinkel, of Park Street, gave a reception to Dr. and Mrs. Stevens, Monday, January 21, 1901, from 3 to 5 o'clock, which was attended by physicians and their wives in large numbers.

---

Dr. J. C. Johnson, of New York, assistant pathologist of Cornell University, read a paper before the Section on Pathology, Buffalo Academy of Medicine, Tuesday evening, January 15, 1901, entitled, sarcoma and sarkoid growth of the skin. Previous to the meeting Dr. Johnson was entertained at dinner at the Saturn Club by Drs. Grover W. Wende and Eugene A. Smith.

DR. EUGENE WASDIN, U. S. Marine Hospital Service, now stationed in Buffalo, has been tendered by the Italian Government the cross of officer of SS. Maurizio et Lazzaro, in recognition of his services in verifying and confirming the Italian studies and discoveries regarding the nature of yellow fever. The permission of congress to accept the decoration doubtless will be granted.

DR. F. W. BARROWS, professor of histology and biology at Buffalo University Medical College, was elected president of the New York State Association of Science Teachers at its recent annual meeting held at Rochester.

DR. WM. D. HAGGARD, Jr., of Nashville, has been elected to fill the chair of gynecology and diseases of children in the medical department of the University of Tennessee, formerly occupied by his father for many years.

DR. N. G. RUSSELL, of Buffalo, has tendered his resignation as physician to the fourth district, to which position he was appointed some months ago, and Dr. H. K. De Groat has been appointed to fill the vacancy.

DR. THOMAS LOTHROP, of Buffalo, whose term as manager of the Buffalo State Hospital expired with the beginning of the year, has been reappointed for a period of four years, by Governor Odell.

DR. CHARLES D. AARON, of Detroit, Mich., U. of B., '91, has been elected clinical professor of diseases of the stomach and intestines in the Detroit College of Medicine, Detroit, Mich.

CAPTAIN HARRY MEAD, M.D., of Buffalo, has tendered his resignation as assistant surgeon of the 65th Regiment, N.Y. National Guard.

DR. CHESTER L. CARLISLE, of Buffalo, has been appointed medical interne at the Manhattan State Hospital, New York.

